# Inherited variants in genes somatically mutated in thyroid cancer

**DOI:** 10.1371/journal.pone.0174995

**Published:** 2017-04-14

**Authors:** Chiara Campo, Aleksandra Köhler, Gisella Figlioli, Rossella Elisei, Cristina Romei, Monica Cipollini, Franco Bambi, Kari Hemminki, Federica Gemignani, Stefano Landi, Asta Försti

**Affiliations:** 1 Division of Molecular Genetic Epidemiology, German Cancer Research Center (DKFZ), Heidelberg, Germany; 2 Department of Biology, University of Pisa, Pisa, Italy; 3 Department of Endocrinology and Metabolism, University of Pisa, Pisa, Italy; 4 Blood Centre, Azienda Ospedaliera Universitaria A. Meyer, Firenze, Italy; 5 Center for Primary Health Care Research, Clinical Research Center, Lund University, Malmö, Sweden; Ohio State University Wexner Medical Center, UNITED STATES

## Abstract

**Background:**

Tumour suppressor genes when mutated in the germline cause various cancers, but they can also be somatically mutated in sporadic tumours. We hypothesized that there may also be cancer-related germline variants in the genes commonly mutated in sporadic well-differentiated thyroid cancer (WDTC).

**Methods:**

We performed a two-stage case-control association study with a total of 2214 cases and 2108 healthy controls from an Italian population. By genotyping 34 single nucleotide polymorphisms (SNPs), we covered a total of 59 missense SNPs and SNPs located in the 5' and 3' untranslated regions (UTRs) of 10 different genes.

**Results:**

The Italian1 series showed a suggestive association for 8 SNPs, from which three were replicated in the Italian2 series. The meta-analysis revealed a study-wide significant association for rs459552 (OR: 0.84, 95%CI: 0.75–0.94) and rs1800900 (OR: 1.15, 95%CI: 1.05–1.27), located in the *APC* and *GNAS* genes, respectively. The *APC* rs459552 is a missense SNP, located in a conserved amino acid position, but without any functional consequences. The *GNAS* rs1800900 is located at a conserved 5'UTR and according to the experimental ENCODE data it may affect promoter and histone marks in different cell types.

**Conclusions:**

The results of this study yield new insights on WDTC, showing that inherited variants in the *APC* and *GNAS* genes can play a role in the etiology of thyroid cancer. Further studies are necessary to better understand the role of the identified SNPs in the development of WDTC and to functionally validate our *in silico* predictions.

## Introduction

Thyroid cancer (TC) is a common endrocrine malignancy. The majority of all thyroid cancers are well-differentiated (WDTC). Among them the most frequent subtype is papillary TC (PTC), followed by follicular TC (FTC); they represent 80–84% and 6–10% of all thyroid carcinomas, respectively [1]. The incidence of TC in the world has been increasing in recent years and it accounts for about 1% of all oncological diseases based on the 2012 GLOBOCAN data [2]. Explanations for the increasing thyroid cancer incidence are controversial [3]. Some experts suggest that the rise in the number of new cancers is due to the increased diagnostic intensity; other experts believe that it is associated to lifestyle changes. Many individual and environmental factors have been considered as risk factors for TC. Indeed, thyroid gland seems to be an organ particularly vulnerable to ionizing radiation and the risk for TC in individuals exposed to radiation at young age persists throughout life [4,5]. Benign nodules/adenomas and goiter seem to have an important role in the predisposition to TC [6], as well as an inherited genetic susceptibility [7,8]. Common genetic variants in low-penetrance genes may interact with each other and with the environment, regulating TC susceptibility [9,10].

Many association studies, both designed candidate gene and genome-wide approaches, have been conducted to identify genetic variants affecting individual susceptibility to WDTC. In particular pathways involving DNA repair (*OGG1*, *XRCC1*, *XRCC2*, *XRCC3*, *ATM*), cell-cycle control (*TP53*), detoxification (*CYP1A1*, *GSTs*) and thyroid function (*TG*) have been intensively studied. Some genetic variants of these genes have been shown to be TC susceptibility factors in humans [11–13]. Genome wide association studies (GWASs) of WDTC have reported associations with single nucleotide polymorphisms (SNPs) at 2q35 (*DIRC3*), 8p12 (*NRG1*), 9q22.33 (*FOXE1*) and 14q13.3 (*NKX2-1*). These genetic risk factors were first identified in Icelandic and European studies, then replicated and confirmed across different populations [14–23]. Moreover, new risk loci at 20q11.22-q12 (*DHX35*) and 14q24.3 (*BATF*) were recently discovered in a GWAS conducted on an Italian population and followed by replication on three different cohorts (Italian, Polish, Spanish) [23]. Other seven loci showed evidence of association with WDTC only among Italians [22,23].

This study aimed to identify novel SNPs involved in WDTC susceptibility, selected in genes somatically mutated in the disease. These genetic variants were analyzed in a two-stage case-control association study. After identification of the candidate genes/polymorphisms associated with the disease, further verification was done by analyzing the functional consequences using *in silico* prediction tools.

## Materials and methods

### Study population

The first Italian case group consisted of 1523 WDTC patients (85% PTC, 11% FTC, 27.4% males, median age 53 years), while the control group comprised 1610 healthy individuals (66.9% males, median age 57 years). The SNPs which showed significant associations (p <0.05) were replicated in a second Italian sample set, totaling 691 affected individuals (100% PTC, 22.4% males, median age 47 years) and 498 controls (23.1% males, median age 53 years), *Table A in* S1 File. The patients were contacted during their routine follow-up by the Department of Endocrinology and Metabolism (University Hospital of Cisanello, Pisa). All patients had a histologically or cytologically confirmed WDTC. The healthy controls were blood donors (University Hospital A. Meyer, Florence) and workers (mainly physicians, nurses and paramedical staff), of the same Hospital of Pisa, without known thyroid disease. The study was authorized by the Pisa University Medical Ethics Committee (CEAVNO, North-West Tuscany Ethical Committee). According to the Helsinki declaration, all individuals signed an informed consent to participate to the study and to allow genetic analyses on their biological samples.

### Gene and SNP selection

The gene selection was done using the browser COSMIC (http://cancer.sanger.ac.uk/cosmic). Starting from the 20 top genes mutated in PTC, we considered those with a mutation frequency higher than 5%, obtaining a total of 11 genes (*APC*, *BRAF*, *CDH1*, *CDKN2A*, *CTNNB1*, *EGFR*, *GNAS*, *IDH1*, *SMAD4*, *TP53*, *TSHR*). We considered all gene transcripts, then selected all missense variants and SNPs located in the 5´UTR and 3´UTR, with a global minor allele frequency (MAF) >10% and MAF >5% in the Italian population. We applied a tagging SNP approach and checked the linkage disequilibrium (LD) of those variants in the Hap Map Project (http://hapmap.ncbi.nlm.nih.gov/) and in the 1000 Genomes Project (http://www.1000genomes.org/). By selecting SNPs with a pair wise LD r^2^<0.8, we reduced the number of SNPs to be genotyped to 34 (5´UTR: 6, 3´UTR: 22 and missense: 6), covering 59 SNPs located in 10 different genes, *Table B in* S1 File. The *BRAF* gene was not further included in our analysis since it did not present any variants fulfilling our selection criteria.

### Genotyping

Genotyping was performed using allele specific PCR based TaqMan Assays (Life Technologies) and KASPar v4.0 Assays (Kbioscience). HOT FIREPol Probe qPCR Mix Plus (ROX) from Solis BioDyne (Tartu, Estonia) was used for the TaqMan Assays, whereas the KASPar genotyping reaction from LGC was prepared according to the KBioscience’s conditions and products (KASP Assay mix, containing the target specific primers, and KASP Master mix). PCR reactions were performed in a 384-well plate format using 5ng DNA in a total reaction volume of 5μl per well. Endpoint genotyping detection was performed using ViiA7 Real-Time PCR System (Applied Biosystems, Weiterstadt, Germany).

### Statistical analysis

The genotype distribution in controls was tested for Hardy-Weinberg equilibrium (HWE) using the chi square (χ2) test. SNPs with a significant deviation from HWE (p<0.005) were excluded from the analysis. Odd ratios (ORs) and 95% confidence intervals (95% CIs) were calculated by logistic regression and adjusted for the covariates age and sex (PROC LOGISTIC, SAS Version 9.2; SAS Institute, Cary, NC). For all SNPs, ORs were calculated according to the allelic model using the major allele as reference. For SNPs with allelic pValue <0.05, we also evaluated the associations according to co-dominant and dominant model using the major homozygote genotype as reference. The pValue <0.05 was considered statistically significant. The power of the study was evaluated using the Quanto Version 1.2 (USC Biostats), assuming a case-control ratio of 1:1 in phase1 and 1:0.7 in phase 2, a baseline risk of 0.001, MAF of 0.1–0.5, OR of 1.0–1.5 according to a log-additive inheritance model and a significance level of 0.05 (2-sided in phase 1; 1-sided in phase 2), *Table C in* S1 File. Furthermore, the meta-analysis of the two Italian sets was conducted. ORs and 95% CIs were calculated using both fixed-effect (Mantel-Haenszel) and random-effect (DerSimonian Laird) models. The significance of overall OR was evaluated by the *Z*-test. Heterogeneity between studies was evaluated with a *Q*-test. Heterogeneity was considered significant when p<0.05. In case of no significant heterogeneity, point estimates and 95% CI were assessed using the fixed-effect model; otherwise, random effects model was employed. Bonferroni´s correction was used, taking into account the number of SNPs involved in the meta-analysis, and the novel statistical significant threshold was 6.25×10^−3^.

### In silico analysis

For each SNP significantly associated with WDTC risk, we carried-out *in silico* analysis to determine its predicted functional effect and to understand the molecular events controlling the gene expression. We performed SIFT and PolyPhen predictions to determine the possible effect of amino acid substitutions on protein function [24]. We used the HaploRegv4.1 software (http://www.broadinstitute.org/mammals/haploreg/haploreg.php) to investigate the influence of the SNPs on gene expression [25]. It uses LD and SNP information from the 1000 Genomes Project to map known genetic variants onto ENCODE, providing a potential mechanism for a SNP influence. ENCODE is a database which visualizes the influence of SNPs based on their effect on chromatin structure, histone modifications, sequence conservation across mammals, regulatory protein binding sites and their effect on regulatory motifs and transcription factor binding sites (TFBSs). This tool includes also an expanded library of expression quantitative trait loci (eQTL) [26]. As a complementary analysis we used the tool RegulomeDB (http://regulomedb.org/) to identify DNA features and regulatory elements that contain the coordinate of the SNP.

## Results

A case-control association study was conducted with patients with sporadic WDTC, mainly PTC, and healthy controls from an Italian population. The two subtypes, papillary and follicular TC, were not analyzed separately due to the small number of FTC (11% of cases). Genotype distribution of three SNPs (rs3864004 in *CTNNB1*; rs2227983 and rs10228436 in *EGFR*) in controls deviated from HWE (p <0.005) and they were excluded from further analyses. The associations according to the allelic model between the SNPs and WDTC are shown in Table 1. The analysis adjusted for the covariates age and sex, revealed a protective role (pValue <0.05) for 5 SNPs: rs459552 within the *APC* gene; rs884904 within *EGFR*; rs8022600 and rs2268477 within *TSHR*; rs7229678 within the *SMDA4* gene. An increased risk of WTDC (pValue <0.05) was found for the rare allele carriers of rs7144481 within *TSHR* gene, and of rs1800900 and rs7121 within the *GNAS* gene.

**Table 1:** Association between genotyped SNPs and WDTC risk in the Italian set1. (*) SNPs with a p-Value_HWE_ <0.005 in controls were excluded from further analyses.

			Unadjusted			Adjusted for sex and age		
Gene	SNP	Minor allele	Allelic OR	95% CI	pValue	Allelic OR	95% CI	pValue
*IDH1*	rs12478635	(T)	0.98	[0.87–1.10]	0.68	0.94	[0.82–1.07]	0.33
CTNNB1*	rs3864004	(A)	0.98	[0.89–1.09]	0.76	0.94	[0.84–1.06]	0.33
*CTNNB1*	rs2953	(G)	0.99	[0.90–1.10]	0.90	0.96	[0.85–1.08]	0.47
*APC*	rs459552	(T)	0.88	[0.78–1.00]	0.05	0.84	[0.73–0.96]	0.01
*APC*	rs41116	(C)	0.98	[0.89–1.09]	0.73	1.02	[0.91–1.15]	0.72
*APC*	rs397768	(G)	0.96	[0.87–1.10]	0.45	0.91	[0.80–1.02]	0.10
EGFR*	rs2227983	(A)	1.00	[0.90–1.12]	0.94	0.96	[0.85–1.09]	0.56
EGFR*	rs10228436	(A)	1.03	[0.93–1.14]	0.61	0.98	[0.88–1.11]	0.79
*EGFR*	rs884225	(C)	0.94	[0.80–1.10]	0.45	0.86	[0.71–1.04]	0.12
*EGFR*	rs884904	(A)	0.89	[0.75–1.06]	0.20	0.82	[0.67–0.99]	0.04
*EGFR*	rs2280653	(G)	1.17	[1.00–1.36]	0.05	1.19	[1.00–1.42]	0.05
*EGFR*	rs940810	(T)	0.99	[0.89–1.10]	0.84	1.02	[0.90–1.16]	0.75
*EGFR*	rs6593211	(A)	1.02	[0.90–1.15]	0.77	1.00	[0.87–1.14]	0.96
*EGFR*	rs34462843	(C)	1.09	[0.96–1.24]	0.18	1.08	[0.93–1.26]	0.29
*TSHR*	rs8022600	(T)	0.87	[0.78–0.96]	5.40×10^−3^	0.82	[0.73–0.92]	1.13×10^−3^
*TSHR*	rs3783941	(C)	0.97	[0.87–1.09]	0.61	1.00	[0.88–1.14]	0.96
*TSHR*	rs1991517	(G)	1.06	[0.88–1.28]	0.53	1.01	[0.81–1.25]	0.96
*TSHR*	rs7144481	(C)	1.12	[0.97–1.31]	0.12	1.20	[1.01–1.43]	0.04
*TSHR*	rs17630128	(C)	1.02	[0.91–1.14]	0.78	1.02	[0.89–1.16]	0.82
*TSHR*	rs2288493	(T)	1.04	[0.91–1.18]	0.60	1.08	[0.92–1.26]	0.34
*TSHR*	rs2288495	(C)	1.01	[0.91–1.12]	0.82	1.01	[0.90–1.14]	0.87
*TSHR*	rs2268477	(A)	0.78	[0.66–0.92]	3.49×10^−3^	0.75	[0.62–0.91]	3.96×10^−3^
*CDH1*	rs1801026	(T)	1.11	[0.96–1.28]	0.16	1.08	[0.91–1.27]	0.37
*CDKN2A*	rs3088440	(A)	0.99	[0.81–1.21]	0.92	0.97	[0.77–1.21]	0.76
*CDKN2A*	rs11515	(C)	1.07	[0.93–1.22]	0.35	1.13	[0.96–1.32]	0.13
*CDKN2A*	rs3731249	(T)	1.04	[0.82–1.33]	0.74	1.12	[0.85–1.48]	0.43
*CDKN2A*	rs2518720	(T)	1.06	[0.95–1.17]	0.31	1.09	[0.96–1.23]	0.17
*TP53*	rs1042522	(G)	0.94	[0.84–1.06]	0.33	0.96	[0.84–1.09]	0.50
*SMAD4*	rs7229678	(C)	0.90	[0.81–1.00]	0.05	0.86	[0.76–0.97]	0.01
*SMAD4*	rs12456284	(G)	0.88	[0.79–0.99]	0.03	0.89	[0.78–1.01]	0.07
*GNAS*	rs1800900	(A)	1.13	[1.02–1.25]	0.02	1.17	[1.04–1.31]	0.01
*GNAS*	rs8125112	(C)	1.07	[0.92–1.25]	0.37	1.09	[0.91–1.29]	0.34
*GNAS*	rs13831	(A)	1.01	[0.91–1.14]	0.79	1.03	[0.90–1.17]	0.66
*GNAS*	rs7121	(C)	1.18	[1.07–1.32]	1.47×10^−3^	1.23	[1.09–1.39]	7.90×10^−4^

SNP: single nucleotide polymorphism, OR: odd ratio, CI: confidence interval

Regarding the associated SNPs (pValue <0.05), we also evaluated the genotype risks in the co-dominant and dominant models. For rs1800900 within the *GNAS* gene, the association of minor homozygote genotype in comparison to the major homozygote genotype was stronger than the association according to the allelic model (pValue = 8.82 x 10^−3^ vs. pValue = 0.01). A dominant model described the association of rs7144481 in *TSHR* with WDTC better than the allelic model (pValue = 0.01 vs. pValue = 0.04).

To validate these findings, we conducted a replication study of the 8 SNPs, statistical significant at the 0.05 level, by genotyping an additional Italian set of 691 cases and 498 controls. Only three SNPs showed ORs in the same direction and at the same level as in the first set (rs459552, rs7144481 and rs1800900). The combined analysis of the two data sets identified two promising variants associated with WDTC risk: rs459552 within *APC*, (OR = 0.84, 95CI% = 0.75–0.94, pValue = 4.00x10^-3^, allelic model) and rs1800900 within *GNAS*, (OR = 1.15, 95CI% = 1.05–1.27, pValue = 4.00x10^-3^, allelic model). All the results are shown in Tables 2 and 3. In summary, the joint analysis detected a decreased risk of WDTC for one SNP (rs459552) in *APC* and a positive association for one SNP (rs1800900) within *GNAS*.

**Table 2:** Association of the 8 top SNPs in the two Italian sets with WDTC risk (Part I).

				Italian1*				Italian2*		Combined	
Gene/SNP		Case	Control	OR [95% CI]	pValue	Case	Control	OR [95% CI]	pValue	OR [95% CI]	pValue
APC (rs459552)	AA	903	908	1.0 [reference]		430	296	1.0 [reference]			
	AT	498	553	0.85[0.71–1.01]	0.07	214	172	0.93[0.71–1.20]	0.56	0.87[0.76–1.01]	0.07
	TT	68	91	0.68[0.47–0.99]	0.04	26	28	0.57[0.32–1.03]	0.06	0.65[0.47–0.88]	6.00 × 10^−3^
	AT + TT	566	644	0.83[0.70–0.99]	0.03	240	200	0.87[0.68–1.12]	0.28	0.84[0.73–0.97]	0.02
	Per allele			0.84[0.73–0.96]	0.01			0.85[0.69–1.05]	0.13	0.84[0.75–0.94]	4.00 × 10^−3^
EGFR (rs884904)	GG	1212	1238	1.0 [reference]		529	404	1.0 [reference]			
	AG	243	287	0.80[0.64–0.99]	0.04	127	87	1.12[0.82–1.53]	0.48	0.89[0.75–1.07]	0.22
	AA	16	16	0.80[0.35–1.82]	0.60	7	3	2.22[0.56–8.80]	0.26	1.05[0.52–2.12]	0.90
	AG + AA	259	303	0.80[0.65–0.98]	0.04	134	90	1.15[0.85–1.57]	0.36	0.90[0.75–1.06]	0.20
	Per allele			0.82[0.67–0.99]	0.04			1.17[0.88–1.56]	0.27	0.92[0.78–1.08]	0.30
TSHR (rs8022600)	GG	464	411	1.0 [reference]		186	167	1.0 [reference]			
	GT	693	744	0.79[0.65–0.95]	0.01	362	237	1.37[1.04–1.80]	0.02	1.03[0.60–1.77]	0.91
	TT	313	363	0.68[0.54–0.86]	1.51 × 10^−3^	131	92	1.17[0.82–1.66]	0.40	0.88[0.52–1.50]	0.63
	GT + TT	1006	1107	0.75[0.63–0.90]	2.44 × 10^−3^	493	392	1.32[1.02–1.71]	0.04	0.99[0.57–1.72]	0.96
	Per allele			0.82[0.73–0.92]	1.13 × 10^−3^			1.12[0.94–1.33]	0.20	0.95[0.70–1.29]	0.75
TSHR (rs7144481)	TT	1090	1189	1.0 [reference]		490	362	1.0 [reference]			
	CT	360	331	1.30[1.07–1.59]	9.17 × 10^−3^	187	123	1.08[0.82–1.42]	0.60	1.22[1.04–1.43]	0.01
	CC	25	29	0.92[0.50–1.71]	0.80	14	11	0.97[0.42–2.22]	0.93	0.94[0.57–1.54]	0.80
	CT + CC	385	360	1.27[1.05–1.54]	0.01	201	134	1.07[0.82–1.39]	0.63	1.20[1.02–1.40]	0.02
	Per allele			1.20[1.01–1.43]	0.04			1.05[0.83–1.33]	0.69	1.14[0.99–1.32]	0.06

SNP: single nucleotide polymorphism, OR: odd ratio, CI: confidence interval

(*) The analysis was conducted adjusting for the covariates of sex and age

**Table 3:** Association of the 8 top SNPs in the two Italian sets with WDTC risk (Part II).

				Italian1*				Italian2*		Combined	
Gene/SNP		Case	Control	OR [95% CI]	pValue	Case	Control	OR [95% CI]	pValue	OR [95% CI]	pValue
APC (rs459552)	AA	903	908	1.0 [reference]		430	296	1.0 [reference]			
	AT	498	553	0.85[0.71–1.01]	0.07	214	172	0.93[0.71–1.20]	0.56	0.87[0.76–1.01]	0.07
	TT	68	91	0.68[0.47–0.99]	0.04	26	28	0.57[0.32–1.03]	0.06	0.65[0.47–0.88]	6.00 × 10^−3^
	AT + TT	566	644	0.83[0.70–0.99]	0.03	240	200	0.87[0.68–1.12]	0.28	0.84[0.73–0.97]	0.02
	Per allele			0.84[0.73–0.96]	0.01			0.85[0.69–1.05]	0.13	0.84[0.75–0.94]	4.00 × 10^−3^
EGFR (rs884904)	GG	1212	1238	1.0 [reference]		529	404	1.0 [reference]			
	AG	243	287	0.80[0.64–0.99]	0.04	127	87	1.12[0.82–1.53]	0.48	0.89[0.75–1.07]	0.22
	AA	16	16	0.80[0.35–1.82]	0.60	7	3	2.22[0.56–8.80]	0.26	1.05[0.52–2.12]	0.90
	AG + AA	259	303	0.80[0.65–0.98]	0.04	134	90	1.15[0.85–1.57]	0.36	0.90[0.75–1.06]	0.20
	Per allele			0.82[0.67–0.99]	0.04			1.17[0.88–1.56]	0.27	0.92[0.78–1.08]	0.30
TSHR (rs8022600)	GG	464	411	1.0 [reference]		186	167	1.0 [reference]			
	GT	693	744	0.79[0.65–0.95]	0.01	362	237	1.37[1.04–1.80]	0.02	1.03[0.60–1.77]	0.91
	TT	313	363	0.68[0.54–0.86]	1.51 × 10^−3^	131	92	1.17[0.82–1.66]	0.40	0.88[0.52–1.50]	0.63
	GT + TT	1006	1107	0.75[0.63–0.90]	2.44 × 10^−3^	493	392	1.32[1.02–1.71]	0.04	0.99[0.57–1.72]	0.96
	Per allele			0.82[0.73–0.92]	1.13 × 10^−3^			1.12[0.94–1.33]	0.20	0.95[0.70–1.29]	0.75
TSHR (rs7144481)	TT	1090	1189	1.0 [reference]		490	362	1.0 [reference]			
	CT	360	331	1.30[1.07–1.59]	9.17 × 10^−3^	187	123	1.08[0.82–1.42]	0.60	1.22[1.04–1.43]	0.01
	CC	25	29	0.92[0.50–1.71]	0.80	14	11	0.97[0.42–2.22]	0.93	0.94[0.57–1.54]	0.80
	CT + CC	385	360	1.27[1.05–1.54]	0.01	201	134	1.07[0.82–1.39]	0.63	1.20[1.02–1.40]	0.02
	Per allele			1.20[1.01–1.43]	0.04			1.05[0.83–1.33]	0.69	1.14[0.99–1.32]	0.06

SNP: single nucleotide polymorphism, OR: odd ratio, CI: confidence interval

(*) The analysis was conducted adjusting for the covariates of sex and age

To explore if the SNPs identified as WDTC risk markers have potential regulatory functions, *in silico* methods were used to predict the effect of amino acid substitutions on protein function and to understand the molecular events controlling gene expression. The results shown in Table 4 summarize all the information obtained by utilizing the HaploRegv4.1 software and RegulomeDB. The *GNAS* gene SNP rs1800900 was identified by SiPhy to be located in a conserved region. As reported by the ENCODE project data, rs1800900 may affect DNAse hypersensitivity sites and enhancer and promoter histone marks in different cell types. Several other linked SNPs show similar functional characteristics. Moreover, Regulome analysis revealed that according to the ChIP-seq data this SNP is involved in GATA1 protein binding sites (data not shown).

**Table 4:** In silico analysis of the predicted regulatory elements in the region surrounding the SNPs in high LD with the 2 most promising variants.

SNP	LD (r^2^)	GENCODE genes	SiPhy cons	Promoter histone marks	Enhancer histone marks	DNAse	Proteins bound	Motifs changed	GRASP QTL hits	Selected eQTL hits
rs34999673	0.84	*GNAS-AS1*		IPSC, BRST, MUS, BLD	ESC, ESDR, BRN, ADRL, HRT, MUS, GI					
rs4812035	0.99	*GNAS-AS1*		MUS	ESDR, ESC, IPSC, ADRL, HRT, MUS, PLCNT, GI			Cdx, Foxa, Foxd3, Foxf1, Foxj1, Foxl1, Foxp1, Hbp1, Mef2, Ncx, Pax4, Pou1f1, Pou3f1, Pou3f2, Pou3f3, Sox		
rs67008625	0.92	*GNAS-AS1*		IPSC, SPLN	ESDR, ESC, IPSC, SKIN, BRN, MUS					
rs3761263	1	*GNAS-AS1*		ESC, ESDR, IPSC, BLD, GI, KID, MUS, PLCNT, LNG, PANC, HRT, SPLN	ESC, ESDR, IPSC, BRST, BLD, SKIN, LIV, BRN, ADRL, PANC, LNG, MUS, GI, THYM, PLCNT, HRT, SPLN, KID	ESC, IPSC, MUS		CEBPB, Sox		
**rs1800900**	**1**	***GNAS***	**✓**	**ESC, ESDR, LNG, IPSC, FAT, STRM, BRST, BLD, MUS, BRN, SKIN, VAS, LIV, GI, ADRL, HRT, KID, PANC, PLCNT, OVRY, SPLN, CRVX, BONE**	**ESC, ESDR, LNG, IPSC, FAT, STRM, BRST, BLD, MUS, BRN, SKIN, LIV, GI, ADRL, PLCNT, THYM, PANC, HRT, SPLN, FAT**	**ESDR, ESC, IPSC, BRST, SKIN,MUS, PLCNT**				**1 hit**
rs1800905	1	*GNAS*		ESC, ESDR, IPSC, STRM, BLD, SKIN, BRN, GI, ADRL, KID, PANC, MUS, PLCNT, HRT, SPLN, FAT, LNG	ESC, ESDR, IPSC, BRST, BLD, STRM, BRN, SKIN, LIV, GI, ADRL, LNG, MUS, PLCNT, THYM, PANC, HRT, SPLN	ESDR, IPSC, SKIN, GI	SUZ12	E2A, Myf, Pou2f2, RXRA, TCF12		2 hits
rs3787497	0.99	*GNAS*		ESC, ESDR, IPSC, STRM, SKIN, ADRL, KID, PANC, MUS, PLCNT, HRT, SPLN, BRN, FAT, GI, LNG	ESC, ESDR, IPSC, BLD, SKIN, GI, ADRL, LNG, MUS, PLCNT, HRT, SPLN	ESC, IPSC, SKIN	SUZ12	BCL, Hic1, NF-kappaB, Nkx2, Roaz		
rs6123832	1	*GNAS*		ESC, ESDR, IPSC, STRM, KID, PANC, MUS, SPLN, BRN, ADLR, HRT, LNG, GI	ESC, ESDR, IPSC, STRM, SKIN, GI, KID, MUS, PLCNT, LNG	ESC, ESDR, IPSC	POL24H8	Ik-2		2 hits
rs6026557	0.91	*GNAS*		ESC, ESDR, IPSC, BLD	ESC, ESDR, IPSC, STRM, ADRL, PANC, MUS, PLCNT			Rad21,TATA	1 hit	
rs56335290	0.81	*6*.*6kb 5' of APC*			ADRL			Arid3a, Pou2f2, Pou3f2		
**rs459552**	**1**	***APC***	**✓**		**BRN**			**Bbx, LRH1**	**3 hits**	**Whole_Blood**

SNP: single nucleotide polymorphism, LD: linkare disequilibrium, eQTL: expression quantitative trait loci

The *APC* gene SNP rs459552 is causing a missense amino acid change (Val1822Asp) in three Ensembl transcripts. However, in all three transcripts it seems to be tolerated by SIFT and considered to be benign by PolyPhen prediction. According to HaploReg v4.1 predictions, this variant alters the binding sites of Bbx and LRH1 transcription factors and it is located in a conserved amino acid position.

## Discussion

To identify new risk variants predisposing to WDTC we investigated genes somatically mutated in the disease for inherited germline polymorphisms, and performed an association study in two case-control sets from Italy. By genotyping of 34 SNPs, we got information of 53 missense SNPs and SNPs located in the 5’ and 3’UTRs of 10 different genes. The Italian1 series showed a suggestive association for 8 SNPs and three of them provided evidence of association in the replication set. Finally, the meta-analysis revealed a study-wide significant association for rs459552 and rs1800900, in the *APC* and *GNAS* genes, respectively.

Although the origin of the samples was the same, there were some differences in the two data sets. First, the Italian1 series was dominated by female cases, while men were the larger group among controls. The Italian2 series was more homogeneous regarding the gender distribution, with a female dominance both among cases and controls. Also, both the cases and controls were older in the first set than in the second one. However, there was no significant difference in allele frequencies between women and men among cases or controls. To avoid sampling biases the analysis was adjusted for age and sex, but the replication series did not have enough statistical power to confirm all the statistically significant results of the first data set. In the first phase, we had an over 80% power to detect an OR of 1.2 for variants with MAF > 20%, however, in the second phase the power was reduced to 56% for variants with MAF of 20%, increasing to 71% for variants with MAF of 50%. As most of the associations were on opposite directions in the two sample sets, they could be considered as false positives in the first sample set. However, the strength of the association of rs459552 (pValue = 4.00x10^-3^) and rs1800900 (pValue = 4.00x10^-3^), together with the in silico data suggests that these two associations may be real.

Our gene selection was based on somatic TC mutation data available at the COSMIC browser. Comparison with the TCGA data showed very similar mutation spectrum, with BRAF being the most commonly mutated gene in PTC [27]. The mutation frequencies in general were, however, usually lower than in COSMIC, also for the genes included in our study. In the current data *APC* is reported to be mutated in 10% of PTC by COSMIC, but only in 0.7% of PTC by TCGA. For *GNAS*, COSMIC reports a 3% mutation frequency, while in TCGA *GNAS* is not included among the top 25 genes.

The Adenomatous Polyposis Coli (*APC*) gene encodes a tumor suppressor protein that acts as an antagonist of the Wnt signaling pathway. It is also involved in other processes including cell migration and adhesion, transcriptional activation, and apoptosis. Defects in this gene cause familial adenomatous polyposis (FAP), an autosomal dominant pre-malignant disease. FAP is an inherited condition caused by germline mutations in the *APC* gene [28]. In addition, somatic mutations of *APC* are observed in sporadic colorectal neoplasm, suggesting that disruption of this tumor suppressor gene may play a role in both familial as well as acquired colorectal tumorigenesis [29]. *APC* has been found to be expressed in normal as well as in neoplastic human thyroid tissue, in which multiple forms of specific RNA transcripts have been detected; it may be hypothesized that alterations in *APC* are likely candidates for a pathogenic role in thyroid tumorigenesis [30]. A study of mutations in *Beta-catenin* and *APC* genes in PTC, FTC and benign thyroid tumors suggested that mutations in these genes are rare and that the activation of the Wnt signaling pathway may not contribute to pathogenesis in human PTC and FTC [31]. However, a better insight into the correlation of *APC* gene mutations on the clinical manifestation of PTC requires further investigation as suggested by a study, which investigated gene variants associated with malignant thyroid disease in FAP. In this patient group carrying an *APC* mutation the prevalence of thyroid malignant diseases was estimated to be 3.7% [32].

In our study, the variant allele carriers of the *APC* SNP rs459552 had a 16% decreased risk of WDTC. The SIFT and PolyPhen predictions did not reveal any damaging effect for this SNP. The effects of *APC* rs459552 substitutions on colorectal cancer (CRC) risk has been investigated but no association between the genetic variant and CRC risk in the Scottish population was observed [33]. Instead, previous studies investigating the possible influence of rs459552 on the association between dietary and lifestyle factors and CRC showed significant results. Indeed, the polymorphism of *APC* gene may have functional significance, especially in the presence of diet, life-style, or other environmental exposures [34]. In a group of Portuguese subjects, a significant interaction was found between the rs459552 polymorphisms and dietary intake of high fat, cholesterol, calcium, and fiber for CRC risk [35].

Our statistical analysis showed a strong association between rs1800900, located in the *GNAS* gene, with WDTC. *GNAS* encodes the α-subunit of the Gs protein (Gsα), which binds GTP and stimulates adenylyl cyclase [36]. Somatic mutations in *Gsα*, and in *TSHR* gene, are the main reason for autonomously functioning, TSH-independent, thyroid nodules in iodine-deficient regions of the world [37,38]. In Gsα, activating mutations result in constitutive activation of the cAMP cascade, which determines clonal expansion of the affected thyroid cells and consequent thyroid autonomy [39]. Studies from Japan reported that somatic mutations of the *Gsα* and *TSHR* genes were caused by point mutations with single amino acid substitutions, which were confined to the hyperfunctioning nodule and which were not presented in neighboring normal thyroid parenchyma [40–42]. No information about rs1800900 has been reported in literature. This SNP seems to be involved in GATA1 protein binding. It has been recognized that GATA1, binding erythroid enhancers of specific genes, acts as a transcriptional activator and it is involved in the differentiation of hematopoietic stem cells to erythrocytes [43]. GATA1 collaborates with BRG1 to disrupt chromatin, regulating the reorganization of nucleosomes at enhancer elements and the binding of transcription factors to those specific DNA sequences [44].

In conclusion, our study provides evidence for the hypothesis that polymorphic variants in the *APC* and *GNAS* genes, which are commonly mutated in sporadic TC, may play a role in the etiology of TC. Further analysis and additional investigations are needed to confirm our results and to validate the accuracy and the specificity of our *in silico* predictions.

## Supporting information

S1 File **Table A. Characteristics of the study population. Table B. SNP selection**. In bold all the SNPs genotyped. SNPs with MAF<0.10 were excluded from the analysis as were the SNPs with a bad sequence (*). From SNPs in high LD (r^2^>0.80; **) only one SNP was genotyped. a) Assay not available. Instead a SNP in high LD (r^2^ = 0.99) was genotyped, rs2518720. **Table C. Statistical power of the TC association study according to Quanto 1.2 power calculation tool**. Based on our study data we used a case-control ratio of 1:1 in phase 1 and 1:0.7 in phase 2, a baseline risk of 0.001, risk allele frequency between 0.1 and 0.5, odds ratio between 1.0 and 1.5, according to a log-additive inheritance model at a significance level of 0.05 (2-sided in phase 1; 1-sided in phase 2).
(DOCX)
