# Correction: Inherited variants in genes somatically mutated in thyroid cancer

**DOI:** 10.1371/journal.pone.0202208

**Published:** 2018-08-07

**Authors:** Chiara Campo, Aleksandra Köhler, Gisella Figlioli, Rossella Elisei, Cristina Romei, Monica Cipollini, Franco Bambi, Kari Hemminki, Federica Gemignani, Stefano Landi, Asta Försti

There is an error in the email address listed for corresponding author Chiara Campo. The email address should be chiaracampo28@gmail.com.

Table 3, “Association of the 8 top SNPs in the two Italian sets with WDTC risk (Part II),” does not appear correctly. Please view Table 3 here.

**Table 3:** Association of the 8 top SNPs in the two Italian sets with WDTC risk (Part II).

		Italian1*				Italian2*				Combined		
Gene/SNP		Case	Control	OR [95% CI]	pValue	Case	Control	OR [95% CI]	pValue	OR [95% CI]	pValue
*TSHR* (rs2268477)											
	CC	1210	1236	1.0 [reference]		566	395	1.0 [reference]			
	AC	248	304	0.79[0.64–0.98]	0.03	115	76	1.19[0.86–1.65]	0.30	0.95[0.64–1.42]	0.81
	AA	10	27	0.42[0.18–0.95]	0.04	9	6	1.30[0.44–3.85]	0.63	0.64[0.33–1.23]	0.18
	AC + AA	258	331	0.76[0.62–0.94]	0.01	124	82	1.20[0.87–1.65]	0.27	0.94[0.60–1.47]	0.78
	Per allele			0.75[0.62–0.91]	3.96 × 10^−3^			1.18[0.88–1.58]	0.26	0.93[0.59–1.44]	0.74
	*SMAD4* (rs7229678)											
GG		566	542	1.0 [reference]		251	187	1.0 [reference]			
CG		651	729	0.84[0.70–1.01]	0.07	337	229	1.05[0.81–1.37]	0.70	0.94[0.78–1.05]	0.19
CC		225	258	0.75[0.58–0.96]	0.02	103	82	0.99[0.69–1.41]	0.93	0.82[0.67–1.01]	0.06
CG + CC		876	987	0.82[0.69–0.97]	0.02	440	311	1.04[0.81–1.33]	0.77	0.88[0.77–1.02]	0.09
Per allele				0.86[0.76–0.97]	0.01			1.01[0.85–1.20]	0.94	0.91[0.82–1.00]	0.06
*GNAS* (rs7121)												
	TT	571	678	1.0 [reference]		269	166	1.0 [reference]			
	CT	651	679	1.19[0.99–1.42]	0.06	322	237	0.81[0.62–1.06]	0.12	0.99[0.68–1.45]	0.98
	CC	240	202	1.51[1.18–1.95]	1.28 × 10^−3^	100	95	0.63[0.44–0.89]	9.63 × 10^−3^	0.98[0.42–2.32]	0.97
	CT + CC	891	881	1.26[1.06–1.49]	7.12 × 10^−3^	422	332	0.76[0.59–0.97]	0.03	0.99[0.60–1.62]	0.96
	Per allele			1.23[1.09–1.39]	7.90 × 10^−4^			0.79[0.66–0.94]	7.26 × 10^−3^	0.99[0.64–1.53]	0.97
	*GNAS* (rs1800900)											
GG		447	491	1.0 [reference]		190	148	1.0 [reference]			
AG		658	744	1.03[0.85–1.25 ]	0.76	354	260	1.04[0.79–1.37]	0.77	1.03[0.88–1.21]	0.69
AA		360	309	1.36[1.08–1.70]	8.82 × 10^−3^	144	90	1.27[0.90–1.81]	0.17	1.33[1.10–1.61]	3.00 × 10^−3^
AG + AA		1018	1053	1.13[0.94–1.34]	0.19	498	350	1.10[0.84–1.43]	0.49	1.12[0.97–1.30]	0.13
Per allele				1.17[1.04–1.31]	0.01			1.11[0.94–1.32]	0.22	1.15[1.05–1.27]	4.00 × 10^−3^

SNP: single nucleotide polymorphism, OR: odd ratio, CI: confidence interval

(*) The analysis was conducted adjusting for the covariates of sex and age
