# Supplementary material for: Inherited variants in genes somatically mutated in thyroid cancer
Source: PLoS One. 2017 Apr 14;12(4):e0174995. doi: 10.1371/journal.pone.0174995 (PMC5391920; doi:10.1371/journal.pone.0174995)
Supplement: S1 File — Table A. Characteristics of the study population. Table B. SNP selection. In bold all the SNPs genotyped. SNPs with MAF<0.10 were excluded from the analysis as were the SNPs with a bad sequence (*). From SNPs in high LD (r2>0.80; **) only one SNP was genotyped. a) Assay not available. Instead a SNP in high LD (r2 = 0.99) was genotyped, rs2518720. Table C. Statistical power of the TC association study according to Quanto 1.2 power calculation tool. Based on our study data we used a case-control ratio of 1:1 in phase 1 and 1:0.7 in phase 2, a baseline risk of 0.001, risk allele frequency between 0.1 and 0.5, odds ratio between 1.0 and 1.5, according to a log-additive inheritance model at a significance level of 0.05 (2-sided in phase 1; 1-sided in phase 2). (DOCX) [file pone.0174995.s001.docx]

**Table A** Characteristics of the study population

| **Italian1** | | **Cases** | **Controls** |
| --- | --- | --- | --- |
| total | | 1523 | 1610 |
| male | | 417 (27.4%) | 1071 (66.9%) |
| female | | 1105 (72.6%) | 531 (32.1%) |
| missing gender | | 1 | 8 |
| median age [range] | | 53 [12-92] | 57 [13-92] |
| histology | | PTC: 1295 – FTC: 169 – Unspecified: 59 |  |
|  |  |  |  |
| **Italian2** | | **Cases** | **Controls** |
| total | | 691 | 498 |
| male | | 155 (22.4%) | 115 (23.1%) |
| female | | 536 (77.6%) | 383 (76.9%) |
| missing gender | | - | - |
| median age [range] | | 47 [18-86] | 53 [16-84] |
| histology | | PTC: 691 |  |
|  |  |  |  |

| **Gene** | **Chr** | **SNP** | **MAF** | **Alleles** | **Position** |
| --- | --- | --- | --- | --- | --- |
| *APC* | 5 | rs79896135* | 0,16 | C/G | 5´UTR |
|  |  | **rs459552** | **0,14** | **A/T** | **coding** |
|  |  | rs201988789* | 0,18 | C/G | coding |
|  |  | rs72541816 | 0,06 | C/G | coding |
|  |  | rs12189 | 0,09 | C/T | 3´UTR |
|  |  | **rs41116**** | **0,47** | **C/T** | **3´UTR** |
|  |  | rs448475** | 0,47 | C/G | 3´UTR |
|  |  | **rs397768** | **0,31** | **A/G** | **3´UTR** |
| *CDH1* | 16 | **rs1801026** | **0,17** | **C/T** | **3´UTR** |
| *CDKN2A* | 9 | rs181996487 | 0,03 | C/A | 3´UTR |
|  |  | **rs3088440** | **0,15** | **G/A** | **3´UTR** |
|  |  | **rs11515** | **0,12** | **C/G** | **3´UTR** |
|  |  | **rs3731249** | **0,01** | **C/T** | **coding** |
|  |  | **rs3814960^a)^** | **0,50** | **C/T** | **5´UTR** |
| *CTNNB1* | 3 | **rs3864004** | **0,35** | **A/G** | **5´UTR** |
|  |  | **rs2953** | **0,36** | **T/G** | **3´UTR** |
| *EGFR* | 7 | rs712829* | 0,26 | G/T | 5´UTR |
|  |  | rs712830* | 0,11 | A/C | 5´UTR |
|  |  | **rs2227983** | **0,30** | **A/G** | **coding** |
|  |  | rs17290225 | 0,02 | G/T/A | 3´UTR |
|  |  | **rs10228436**** | **0,37** | **A/G** | **3´UTR** |
|  |  | rs10277413** | 0,37 | G/T | 3´UTR |
|  |  | **rs884225** | **0,20** | **T/C** | **3´UTR** |
|  |  | **rs884904**** | **0,20** | **A/G** | **3´UTR** |
|  |  | rs940811 | 0.06 | A/G | 3´UTR |
|  |  | **rs2280653** | **0,24** | **A/G** | **3´UTR** |
|  |  | rs884419** | 0.20 | A/G | 3´UTR |
|  |  | **rs940810** | **0,16** | **C/T** | **3´UTR** |
|  |  | rs3809826* | 0.29 | A/G | 3ÚTR |
|  |  | **rs6593211**** | **0,49** | **A/G** | **3´UTR** |

| **Gene** | **Chr** | **SNP** | **MAF** | **Alleles** | **Position** |
| --- | --- | --- | --- | --- | --- |
| *EGFR* | 7 | rs7334** | 0.49 | C/A | 3´UTR |
|  |  | rs1357973 | 0.06 | C/A | 3´UTR |
|  |  | **rs34462843**** | **0.28** | **A/C** | **3´UTR** |
|  |  | rs35891645** | 0.31 | C/T | 3´UTR |
| *GNAS* | 20 | **rs1800900** | **0,39** | **A/G** | **5´UTR** |
|  |  | rs61749697 | 0,02 | C/T | coding |
|  |  | rs61749698 | 0,07 | A/C | coding |
|  |  | **rs7121** | **0,39** | **C/T** | **3´UTR** |
|  |  | **rs8125112** | **0,15** | **C/T** | **3´UTR** |
|  |  | **rs13831** | **0,23** | **A/G** | **3´UTR** |
|  |  | rs8620 | 0,06 | C/T | 3´UTR |
| *IDH1* | 2 | **rs12478635** | 0,25 | G/T | **5´UTR** |
|  |  | rs34218846 | 0,04 | C/T | coding |
| *SMAD4* | 18 | **rs12456284** | **0,23** | **A/G** | **3´UTR** |
|  |  | rs3819122** | 0,41 | A/C | 3´UTR |
|  |  | **rs7229678**** | 0,41 | C/G | coding |
|  |  | rs2282544 | 0,05 | T/C | 3´UTR |
| *TP53* | 17 | rs17884306 | 0,05 | C/T | 3´UTR |
|  |  | **rs1042522** | **0,4** | **C/G** | **coding** |
| *TSHR* | 14 | rs2234919 | 0,03 | A/C | coding |
|  |  | **rs3783941** | **0,42** | **A/C** | **coding** |
|  |  | **rs1991517** | **0,11** | **C/G** | **coding** |
|  |  | **rs2268477** | **0,22** | **A/C** | **3´UTR** |
|  |  | **rs7144481** | **0,16** | **C/T** | **3´UTR** |
|  |  | **rs17630128** | **0,27** | **C/T** | **3´UTR** |
|  |  | **rs2288493** | **0,18** | **C/T** | **3´UTR** |
|  |  | **rs2288495**** | **0,33** | **G/C** | **3´UTR** |
|  |  | **rs8022600** | **0,46** | **G/T** | **5´UTR** |
|  |  | rs2288496** | 0,33 | T/C | 3´UTR |

**Table B** SNP selection

In bold all the SNPs genotyped. SNPs with MAF<0.10 were excluded from the analysis as were the SNPs with a bad sequence (*). From SNPs in high LD (r^2^>0.80; **) only one SNP was genotyped. a) Assay not available. Instead a SNP in high LD (r^2^=0.99) was genotyped, rs2518720

b)

a)

Chr: chromosome, SNP: single nucleotide polymorphism, MAF: minor allele frequency

**Table C** Statistical power of the TC association study according to Quanto 1.2 power calculation tool. Based on our study data we used a case-control ratio of 1:1 in phase 1 and 1:0.7 in phase 2, a baseline risk of 0.001, risk allele frequency between 0.1 and 0.5, odds ratio between 1.0 and 1.5 according to a log-additive inheritance model at a significance level of 0.05 (2-sided in phase 1; 1-sided in phase 2).

| **Allele frequency** | **Odds ratio** | **Power in phase 1** | **Power in phase 2** | **Baseline risk** |
| --- | --- | --- | --- | --- |
| 0.1 | 1.0 | 0.05 | 0.05 | 0.0010 |
|  | 1.1 | 0.20 | 0.17 | 0.0010 |
|  | 1.2 | 0.59 | 0.39 | 0.0010 |
|  | 1.3 | 0.89 | 0.63 | 0.0011 |
|  | 1.4 | 0.99 | 0.82 | 0.0011 |
|  | 1.5 | 1.00 | 0.93 | 0.0011 |
| 0.2 | 1.0 | 0.05 | 0.05 | 0.0010 |
|  | 1.1 | 0.32 | 0.24 | 0.0011 |
|  | 1.2 | 0.83 | 0.56 | 0.0011 |
|  | 1.3 | 0.99 | 0.83 | 0.0012 |
|  | 1.4 | 1.00 | 0.96 | 0.0012 |
|  | 1.5 | 1.00 | 0.99 | 0.0013 |
| 0.3 | 1.0 | 0.05 | 0.05 | 0.0010 |
|  | 1.1 | 0.40 | 0.28 | 0.0011 |
|  | 1.2 | 0.91 | 0.65 | 0.0012 |
|  | 1.3 | 1.00 | 0.90 | 0.0012 |
|  | 1.4 | 1.00 | 0.98 | 0.0013 |
|  | 1.5 | 1.00 | 1.00 | 0.0014 |
| 0.4 | 1.0 | 0.05 | 0.05 | 0.0010 |
|  | 1.1 | 0.44 | 0.30 | 0.0011 |
|  | 1.2 | 0.94 | 0.70 | 0.0012 |
|  | 1.3 | 1.00 | 0.93 | 0.0012 |
|  | 1.4 | 1.00 | 0.99 | 0.0013 |
|  | 1.5 | 1.00 | 1.00 | 0.0014 |
| 0.5 | 1.0 | 0.05 | 0.05 | 0.0010 |
|  | 1.1 | 0.45 | 0.31 | 0.0011 |
|  | 1.2 | 0.94 | 0.70 | 0.0012 |
|  | 1.3 | 1.00 | 0.93 | 0.0013 |
|  | 1.4 | 1.00 | 0.99 | 0.0014 |
|  | 1.5 | 1.00 | 1.00 | 0.0016 |
